# Renin cells orchestrate a neuro-endocrine microenvironment of the kidney arterial tree in health and disease

**DOI:** 10.1172/jci.insight.197709

**Published:** 2026-07-16

**Authors:** Manako Yamaguchi, Georgina Gyarmati, Liam McLaughlin, Hiroki Yamaguchi, Jason P. Smith, Lucas Ferreira de Almeida, Daisuke Matsuoka, Alexandre G. Martini, Sara M. Wilmsen, Sijie Hao, Kazuki Tainaka, Silvia Medrano, Sanjay Jain, Janos Peti-Peterdi, Maria Luisa S. Sequeira-Lopez, R. Ariel Gomez

**Affiliations:** 1 Department of Pediatrics Child Health Research Center, University of Virginia School of Medicine, Charlottesville, Virginia, USA.; 2 Department of Physiology and Neuroscience and Department of Medicine, Zilkha Neurogenetic Institute, University of Southern California, Los Angeles, California, USA.; 3 Department of Medicine, Washington University School of Medicine, St. Louis, Missouri, USA.; 4 Laboratory of Molecular Pharmacology, Faculty of Health Sciences, University of Brasília, Brasília, Brazil.; 5 Molecular Electron Microscopy Core and; 6 Advanced Microscopy Facility, University of Virginia School of Medicine, Charlottesville, Virginia, USA.; 7 Department of System Pathology for Neurological Disorders, Brain Research Institute, Niigata University, Niigata, Japan.; 8 Department of Pediatrics, Washington University School of Medicine, St. Louis, Missouri, USA.

**Keywords:** Development, Nephrology, Vascular biology, Angiogenesis, Transcriptomics

## Abstract

Renin cells are essential for survival and serve as key regulators of blood pressure and fluid-electrolyte homeostasis. Their function and identity are dependent on signals from their local microenvironment afforded by neighboring cells and nerves. Whether and how renin cells contribute to the development and maintenance of this microenvironment remains unclear. Because renin cells are rare — 0.01 % of kidney cells — conventional histological approaches cannot capture their interaction with nerve fibers and surrounding cells within the nephron and its vasculature. Using high-resolution 3D imaging, cell-specific multicolor reporter mice, single-cell RNA-seq, and conditional gene deletions, we mapped how renin cells assemble within arterioles and communicate with axon fibers to organize the growth and orientation of the kidney arterioles during development and disease. This coinductive process is mediated by *Ngf* produced by renin cell precursors and is necessary for renin cell survival and innervation. Interestingly, renin enzymatic insufficiency elevates *Ngf* and drives arteriolar hypertrophy with aberrant axon sprouting and hyperinnervation. These findings indicate that renin cells regulate kidney neurovascular development, revealing them as active organizers of their local neuroregulatory microenvironment in health and disease.

## Abstract 2

Renin cells closely interact with nerves to orchestrate renal neurovascular development, and their overstimulation during renin enzymatic deficiency drives abnormal renal vascular remodeling and hyperinnervation.

## Graphical abstract

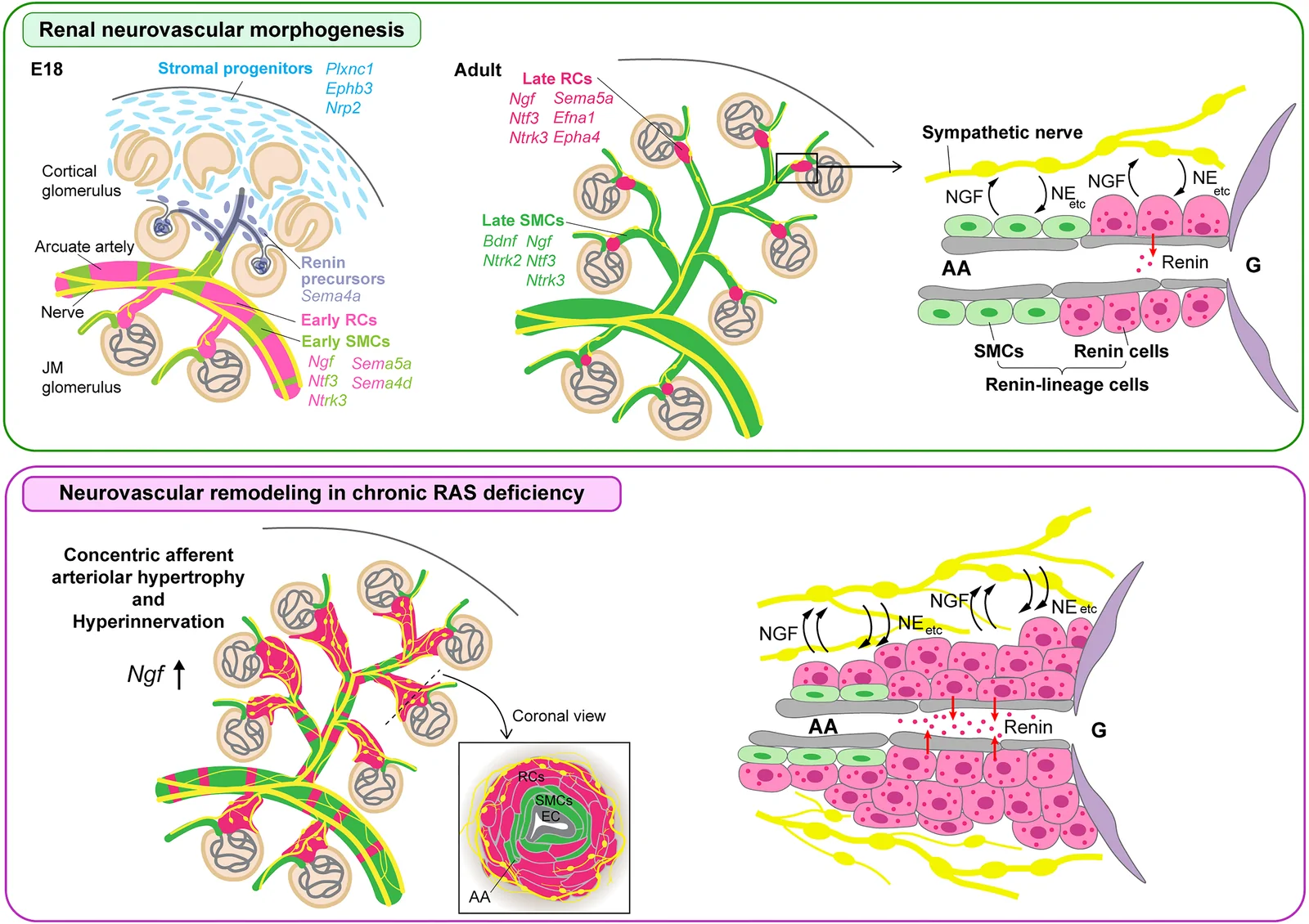

## Introduction

Renin cells appeared in nature over 400 million years ago, remaining essential for survival throughout evolution (1). They control blood pressure and fluid-electrolyte homeostasis by releasing renin from juxtaglomerular (JG) cells located at the tip of the afferent arterioles (AAs) at the entrance to the glomeruli. The release of renin by JG cells is tightly regulated by their intrinsic ability to sense changes in perfusion pressure, sympathetic fibers, and signals from the macula densa, which convey the status — volume and composition — of the extracellular fluid (1, 2). However, it is unknown how the JG microenvironment is generated, how renin cells are innervated, and whether renin cells and nerve fibers cooperate to form a functional renal arterial tree essential for extrauterine life and the maintenance of homeostasis. This knowledge is of fundamental biological and medical importance, given that 1.3 billion people are hypertensive and usually treated with renin-angiotensin-aldosterone-system (RAAS) inhibitors (3–6). Unfortunately, the long-term effects of these inhibitors on renin cells have received little attention. Emerging evidence indicates that chronic RAAS inhibition overactivates renin cells, which drive a clinically silent yet progressive and insidious, arteriolar remodeling of the kidney vasculature that occurs in every species examined thus far, including humans (7–12). This remodeling takes the form of concentric arteriolar hypertrophy, in which renin cells undergo a phenotypic transformation into an embryonic, secretory, and invasive state and induce smooth muscle cells (SMCs) to accumulate concentrically and recruit sympathetic nerve fibers (2, 7–10). The fact that arterial hypertrophy does not occur in renin cell–ablated models (13) indicates that renin cells themselves orchestrate this process. Together, these findings suggest the capacity of renin cells to organize their local environment. However, the spatial architecture linking renin cells to their innervation across developmental, adult, and disease states, and the functional roles of renin cells in establishing and maintaining this innervation are unknown. This knowledge gap represents a major limitation in our ability to properly evaluate long-term efficacy and safety of RAAS-inhibitory therapies and understand the mechanisms of medical renal denervation, which has recently attracted renewed attention for the management of intractable hypertension (14).

We used high-resolution 3-dimensional (3D) imaging, single-cell RNA seq, ultrabright renin cell reporter mice, and in vivo conditional gene deletions to define the spatiotemporal link between renin cell distribution and their innervation. Results show that renin cells produce neurotrophins, including nerve growth factor (NGF), which ensures appropriate renin cell–axon fibers functional and structural development. Renin cells and nerve fibers coinduce each other and regulate kidney neurovascular development, revealing them as active organizers of the local neuroregulatory microenvironment in health and disease.

## Results

### Three-dimensional organization and distinct cellular architecture of renin cells within renal AAs.

**Figure 1:**
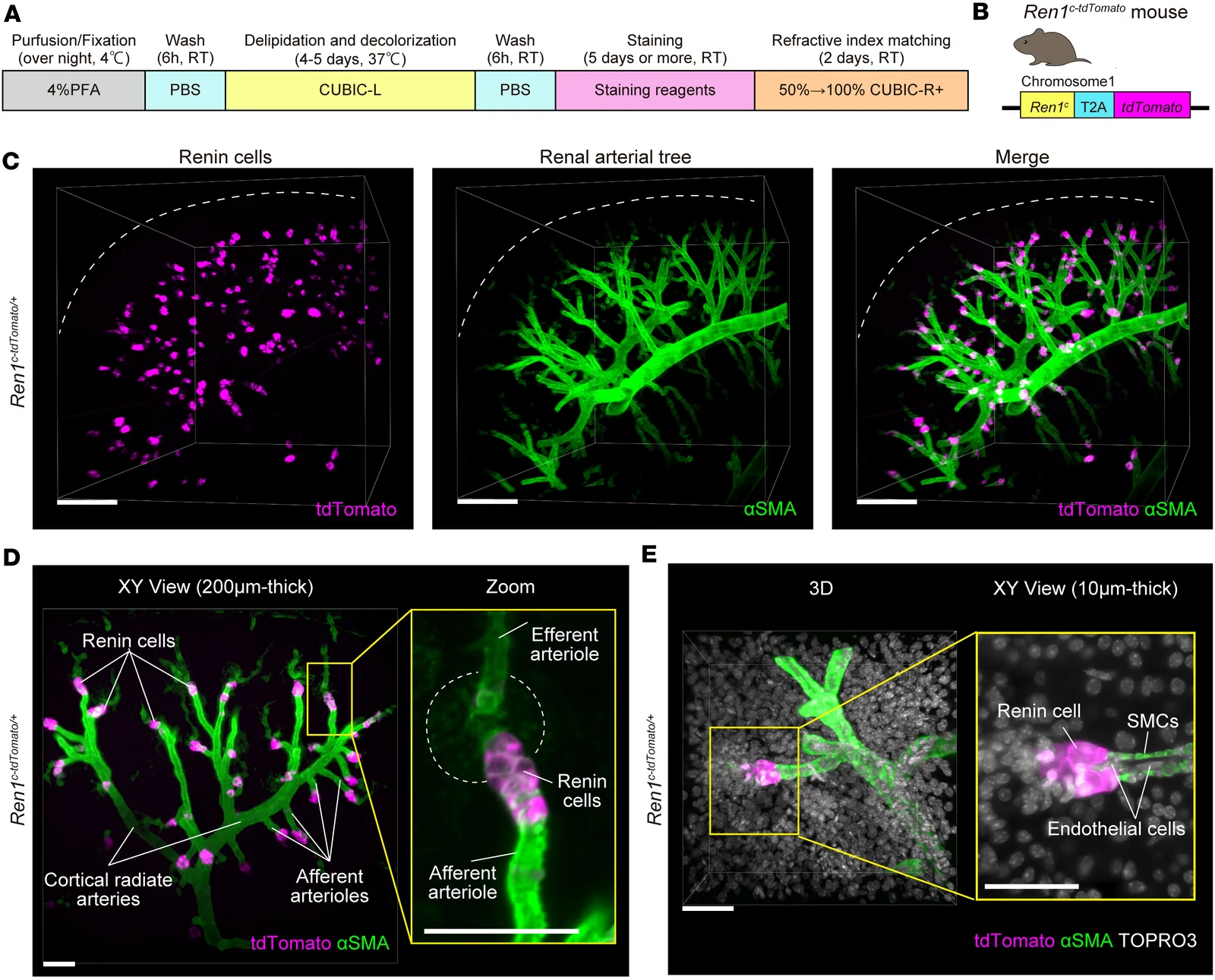
Tissue clearing–based 3D imaging of *Ren1^c-tdTomato/+^* mice allow high-resolution 3D structural observations of renin cells and vascular tree. (**A**) Workflow for kidney clearing and immunostaining (CUBIC protocol). (**B**) *Ren1^c-tdTomato^* mouse generation schematic. (**C**) Three-dimensional visualization of renin cells (tdTomato) and renal arterial tree within the kidney cortex of a 7-month-old *Ren1^c-tdTomato/+^* mouse. Dashed lines: kidney surface. Scale bars: 300 μm. (**D**) XY-plane (200 μm thickness) showing detailed structure of peripheral renal arterial tree (green) and localization of renin cells. Right panel: Cell-level detail. Dashed circle indicates glomeruli. Scale bars: 100 μm. (**E**) High-resolution 3D image depicting spatial relationships between renin cells, SMCs, and nuclei in the kidney cortex of a *Ren1^c-tdTomato/+^* mouse. Right panel: Enlarged XY-plane view (10 μm thickness) illustrating spatial relationships among renin cells, endothelial cells, and SMCs. Scale bars: 50 μm. RT, room temperature; αSMA, α-smooth muscle actin. See also Supplemental Videos 1 and 2.

Renin cells exhibit extraordinarily adaptive plasticity throughout life, altering their morphology, distribution, number, and secretory state in response to physiological demands (1, 2). To elucidate the spatiotemporal dynamics of renin cell distribution within the renal vasculature, we employed 3D imaging combined with immunostaining and a *Ren1c*^-tdTomato^–knock-in reporter mouse line, which provides an ultrabright endogenous signal that specifically labels renin-expressing cells (15) (Figure 1, A and B). Heterozygous (*Ren1^c-tdTomato/+^*) mice have normal renal phenotypes, renin activity, and blood pressure (15). Using *Ren1^c-tdTomato/+^* mice, we show the precise distribution of individual renin cells and their relationship with mural cells within the 3D architecture of the renal arterial tree at single-cell resolution (Figure 1, C and D, and Supplemental Video 1; supplemental material available online with this article; https://doi.org/10.1172/jci.insight.197709DS1). Renin cells in adult mice were localized exclusively at the glomerular inlet near the terminal segments of the AAs.

Staining with TO-PRO-3 iodide (TOPRO3) showed nuclei, cellular shape and spatial arrangement of vascular SMCs, renin cells, and their relationship with endothelial cells (Figure 1E and Supplemental Video 2). In renal arterioles, endothelial cells were oriented horizontally, aligning their nuclei along the longitudinal axis of the blood vessel lumen. In contrast, SMCs formed a single layer arranged perpendicularly around the endothelial layer, constituting the mural vessel wall. Although continuous with SMCs, renin cells exhibited a distinct rounded myoepithelioid morphology, with their nuclei positioned eccentrically toward the cell periphery. Renin cells formed characteristic raspberry-like clusters, and endothelial cells lined the luminal surface of these clusters. Notably, endothelial cells were less densely packed compared with SMCs and renin cells, potentially facilitating efficient transmission of blood flow– and pressure-related mechanical signals from individual endothelial cells to multiple surrounding SMCs and renin cells.

### During physiological stress, renin cells swell and increase in numbers, and SMCs along the AAs switch to the renin phenotype.

**Figure 2:**
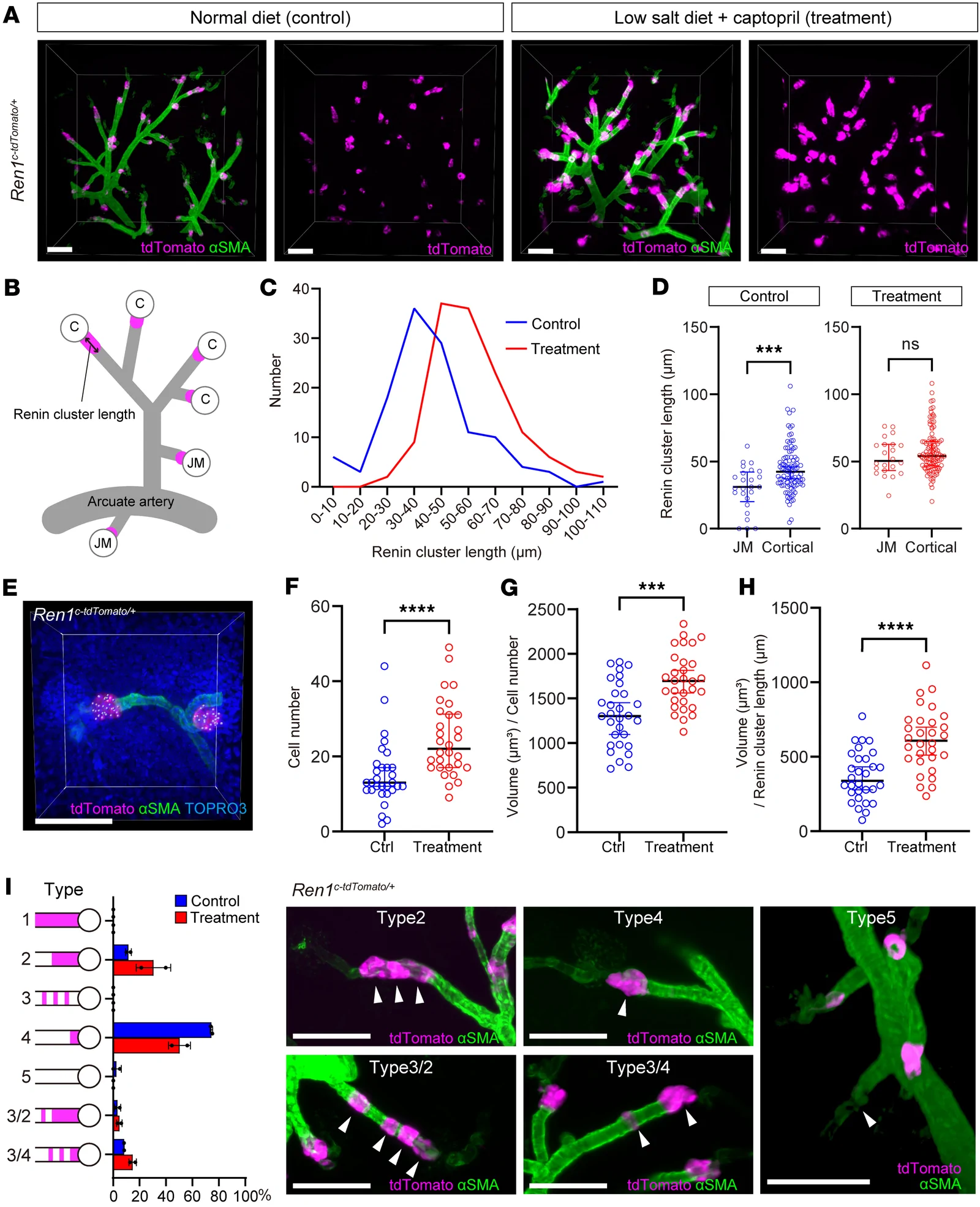
Acute physiological stress markedly alters the number, volume, and distribution of renin cells in AAs. (**A**) Representative 3D images of kidney cortex from 3-month-old *Ren1^c-tdTomato/+^* mice (control versus low-salt diet + captopril). Under physiological stress, renin cells exhibited enhanced brightness, swelled in JG regions, and extended upstream along the AAs, indicative of recruitment. Scale bars: 100 μm. (**B**) Schematic of cortical (**C**) and juxtamedullary (JM) AA structure. Renin cluster length was defined as the longitudinal extension of renin cells along the AA from the glomerular inlet. (**C**) Histogram showing elongated renin cluster lengths under physiological stress. (**D**) Renin clusters were shorter in JMAAs than cortical AAs in controls (*P* = 0.0009), but not under physiological stress (*P* = 0.3287). Data from 121 control and 129 treated AAs (2 mice/group; Mann-Whitney *U* test). (**E**) Three-dimensional reconstruction of renin cell nuclei (Imaris spot). Scale bar: 100 μm. (**F**–**H**) Cell number (**F**), cluster volume per cell (**G**), and volume per length (**H**) significantly increased by treatment. Data from 30 AAs/group (2 mice/group; Mann-Whitney *U* test). (**I**) Left panel: Classification and frequency of renin localization types. Data represent measurements from 2 mice per group. Right panels: Representative 3D images illustrating each type. White arrowheads indicate renin cell localization patterns. Scale bars: 100 μm. ***P < 0.001; ****P < 0.0001. Ctrl, control. See also Supplemental Figure 1.

Under physiological stress, such as volume depletion, hypotension, or inhibition of the RAAS, SMCs along renal arterioles are transformed (and thus recruited) to synthesize and release renin (1, 2). To visualize changes in the distribution of renin cells following a short-term physiological stress, we treated a group of 3-month-old *Ren1^c-tdTomato/+^* mice with captopril and low-sodium diet for 8 days (16). The intensity of tdTomato fluorescence in renin cells markedly increased, and renin cell clusters enlarged in comparison with controls (Figure 2A). To quantitate these changes, we examined a total of 121 control and 129 treated AAs from the renal arterial trees from 2 mice per group. In each arteriole, we confirmed 3D continuity from the arcuate artery to the glomerulus and measured the continuous length of the tdTomato-expressing segment (renin cluster length) extending from the glomerulus upstream the arteriole (Figure 2B). The histogram in Figure 2C clearly illustrates that the renin cluster length increased upon treatment. Indeed, the median renin cluster length was significantly longer in treated animals compared with controls (Supplemental Figure 1A). We further classified the AAs into cortical and juxtamedullary groups based on their branching location (Figure 2B). Under control conditions, cortical AAs exhibited significantly longer renin cluster lengths compared with juxtamedullary AAs (*P* = 0.001), but no significant difference was observed between these 2 groups after short-term treatment with captopril and low-sodium diet (Figure 2D).

Next, to determine whether the enlargement of renin clusters was due to an increase in cell number or cellular hypertrophy, we performed 3D nuclear staining analyses (Figure 2E). We examined 30 control and 30 treated renin clusters from cortical AAs and measured their volume, length, and the number of cells per cluster. The treatment significantly increased the number of renin cells compared with controls (Figure 2F). In addition, the ratios of cluster volume to cell number and cluster volume to cluster length were significantly higher in treated mice compared with controls (Figure 2, G and H). These findings indicate that enlargement of each renin cluster in response to short-term physiological stress is due to increased cell number and individual cell hypertrophy. Moreover, in treated animals, numerous AAs exhibited a distinct recruitment pattern, characterized by tdTomato fluorescence extending longitudinally along the arteriole walls in a stripe-like manner, indicating newly recruited renin cells (Figure 2A). We classified the localization patterns of renin cells within AAs into 7 previously defined types based on immunostaining data from microdissected rat renal arteries (17). Applying this classification to our dataset (*n* = 121 controls, *n* = 129 treated from 2 mice per group), we identified 5 types, excluding types 1 and 3 (Figure 2I). In control kidneys, 74.4% of AAs were classified as Type 4, characterized by renin expression strictly confined to the JG region (Figure 2I). In contrast, under short-term physiological stress, the proportion of Type 4 AAs decreased to 50.3%, accompanied by increases in other types indicative of expanded renin expression. For instance, Type 2, in which clusters of renin cells extend proximally from the glomeruli but do not cover the entire length, increased from 11.5% in the control group to 30.4% after treatment (Figure 2I). Type 3/2 or 3/4, a mixed type combining types 2 or 4 with a striped distribution pattern (Type 3), increased from 11.5% to 19.3% (Figure 2I). In addition, Type 5 arterioles (lacking renin expression) accounted for 2.5% in controls and were completely absent following treatment (Figure 2I). The renin^+^ length of AAs, including the striped distribution pattern, was significantly longer in the treatment group, with 14.7% exceeding 100 μm (Supplemental Figure 1, B–D). In summary, detailed 3D images revealed morphological changes in renin cells induced by short-term physiological stress, including clear alterations in the size, cell number, and spatial distribution of renin cell clusters within the renal arterial tree.

### Sympathetic and sensory nerve fibers establish neuroeffector synapses with renin cells.

**Figure 3:**
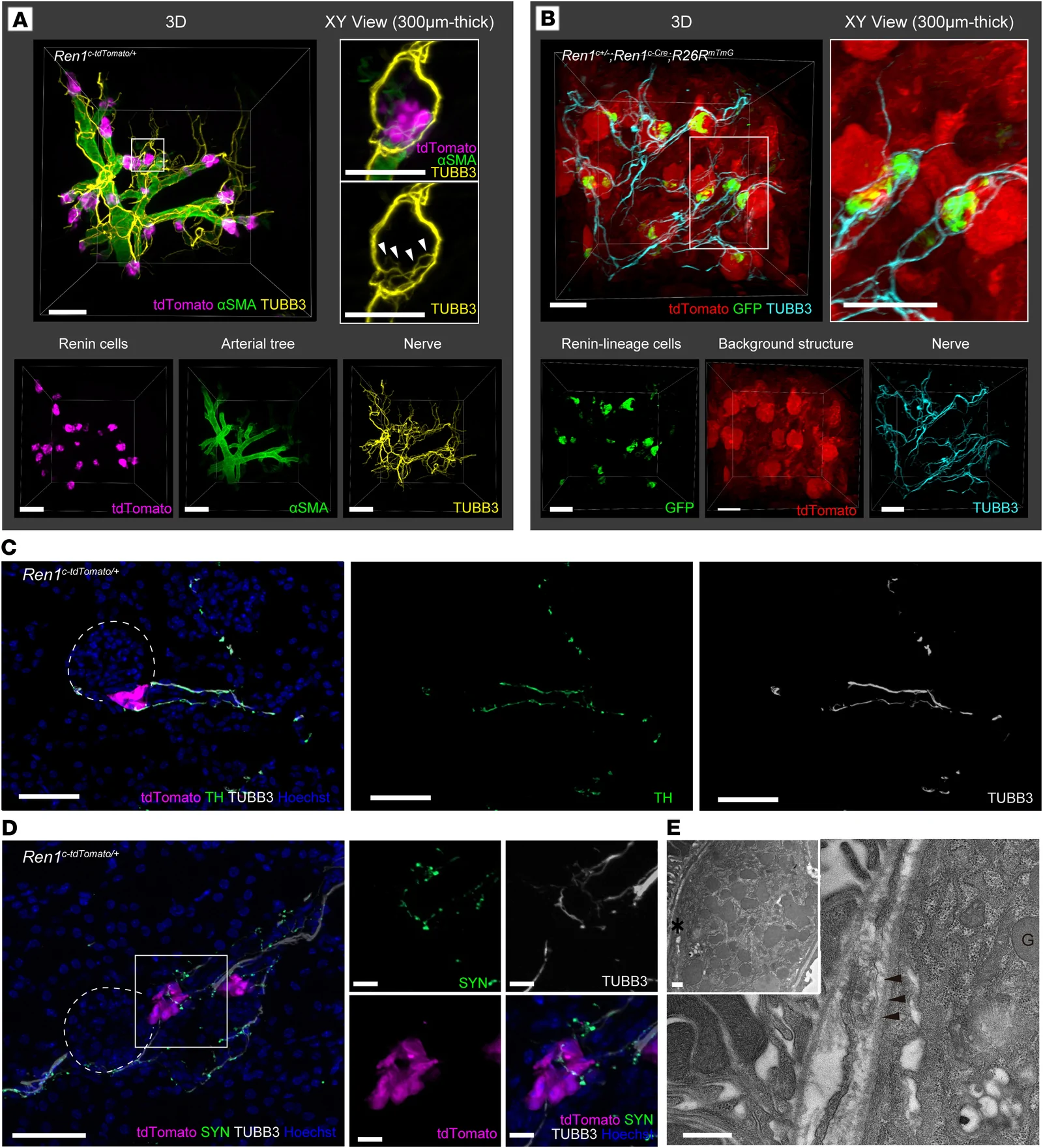
Dense sympathetic innervation of renin cell clusters in AAs. (**A**) Three-dimensional images of *Ren1^c-tdTomato/+^* mouse kidney cortex showing renin cells, arterial tree, and nerve fibers. The enlarged XY view (right panels) highlights nerve fibers enveloping the renin cells, with fine branching of nerve fibers indicated by arrowheads. Bottom panels depict separate channels. Scale bars: 100 μm (left and bottom panels); 50 μm (enlarged view). (**B**) Three-dimensional images from *Ren1^c+/–^; Ren1^c-Cre^; R26R^mTmG^* mice labeled for renin-lineage cells (GFP), other tissue structures (tdTomato), and nerve fibers. Enlarged XY view highlights nerve fiber arrangement along afferent and efferent arterioles, avoiding glomeruli. Bottom panels depict separate channels. Scale bars: 100 μm. (**C**) Immunofluorescence staining of *Ren1^c-tdTomato/+^* mouse kidneys for TH and TUBB3, confirming sympathetic innervation of renin cells. Dashed circle: glomerulus. Scale bars: 50 μm. (**D**) Synaptic marker synaptophysin (SYN) colocalized with TUBB3^+^ nerve fibers, suggesting synaptic connections with renin cells. Right panels: Enlarged views showing colocalization. Dashed circle indicates glomerulus. Scale bars: 50 μm (enlarged view); 10 μm (right enlarged panels). (**E**) Electron microscopy image illustrating nerve terminal containing synaptic vesicles (arrowhead), closely associated with a renin-secreting cell containing renin granules (labeled G). Inset: low-magnification view of a renin cell. The main panel is an enlarged view of the region indicated by an asterisk (*). Scale bars: 500 nm. See also Supplemental Figure 2 and Supplemental Videos 3 and 4.

To define how nerve fibers innervate renin cells, we performed 3D immunostaining with a fluorescently labeled anti–tubulin β 3 (TUBB3) antibody, a pan-neuronal marker. In the adult *Ren1^c-tdTomato/+^* mouse kidney cortex, nerve fibers branched along the arterial tree and, upon reaching renin cells, emitted thin axonal branches that intimately intertwined with renin cells (Figure 3A and Supplemental Video 3). After making close contact with renin cells, axons continued to extend toward the glomerulus. To precisely visualize the 3D relationship between nerve fibers and the glomeruli, we also performed TUBB3 staining in *Ren1^c+/–^; Ren1^c-Cre^; R26R^mTmG^*, in which renin lineage cells are labeled with GFP and all other cells express tdTomato (8). The visceral layer of renal corpuscles strongly expressed tdTomato, clearly outlining the glomerular structure. Interestingly, after reaching the renin cells, axons did not penetrate into the glomerulus, where intraglomerular MCs reside. Instead, axons passed through the JG apparatus (JGA), extended along efferent arterioles, and further branched toward peripheral segments of the arterioles (Figure 3B and Supplemental Video 4). We next identified which types of axons innervated renin cells and the vasculature. Tyrosine hydroxylase (TH) immunostaining confirmed that the majority of fibers contacting renin cells were sympathetic nerve fibers (Figure 3C). Additionally, calcitonin gene-related peptide–positive (CGRP^+^) thin sensory nerve fibers were also observed in close proximity to the renin cell clusters (Supplemental Figure 2). Furthermore, immunostaining with the synaptic marker synaptophysin confirmed the presence of synaptic vesicles along the nerve fibers associated with renin cells, indicating that “en passant” neuroeffector junctions are formed at these sites (Figure 3D). Consistent with these observations, electron microscopy revealed axon varicosities containing synaptic vesicles closely apposed to renin-secreting granular cells, further supporting the direct autonomic innervation of renin cells (Figure 3E).

### Spatiotemporal organization of renin cells and axonal growth trajectories across the renal arterial tree.

**Figure 4:**
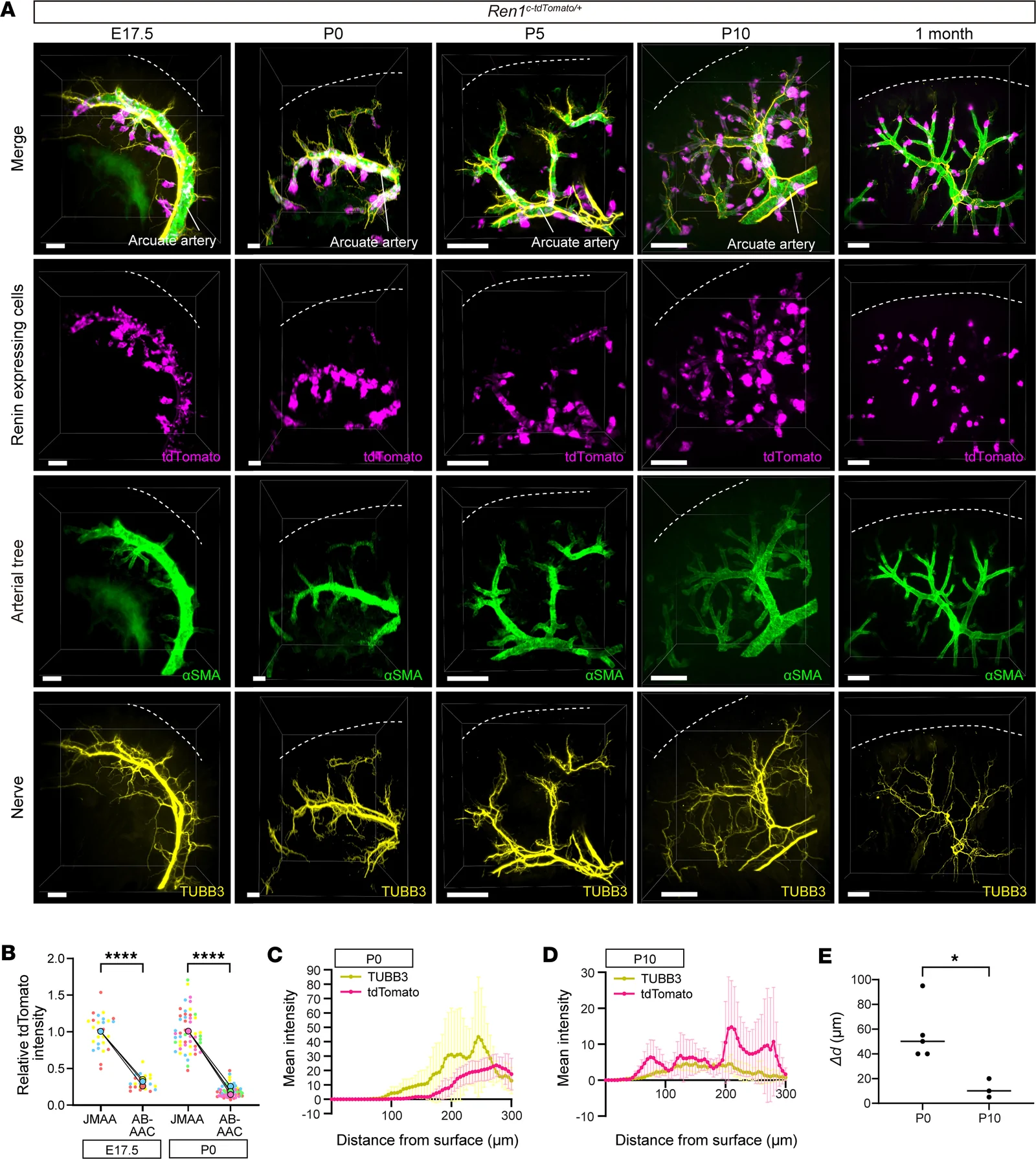
Spatiotemporal organization of renin cells and axonal growth trajectories across the renal arterial tree during kidney development. (**A**) Three-dimensional views of renin expressing cells, arterial tree, and nerves at E17.5, P0, P5, P10, and 1 month in *Ren1^c-tdTomato/+^* mice. Dashed lines indicate kidney surface. Scale bars: 100 μm. (**B**) Relative tdTomato intensity in juxtamedullary afferent arterioles (JMAA) and arterial branches from arcuate arteries to the outer cortex (AB-AAC) at E17.5 (*n* = 3) and P0 (*n* = 5) (linear mixed-effects model). Small colored circles indicate individual measurements, and large circles indicate the mean for each sample. (**C**–**E**) Distance-dependent distribution of TUBB3 and tdTomato signals from the kidney surface at P0 (*n* = 5) and P10 (*n* = 3). (**C** and **D**) Mean intensity profiles of TUBB3 and tdTomato. Solid lines indicate the mean across mice, and shaded areas represent mean ± SD. At P0, TUBB3 intensity rises closer to the surface than tdTomato, whereas at P10 the onset of TUBB3 and tdTomato signals is nearly coincident. (**E**) *Δd*, defined as the difference in onset distance between tdTomato and TUBB3 (*Δd* = d_onset[tdTomato] − d_onset[TUBB3]), is larger at P0 than at P10 (Mann-Whitney *U* test). *P < 0.05, ****P < 0.0001. See also Supplemental Figure 3 and Supplemental Video 5.

During development, renin precursor cells play a crucial role in the morphogenesis and branching of the developing renal arterial tree (18). Since mouse glomerulogenesis continues until approximately 7–10 days after birth, the neonatal renal cortex contains nephrons at various developmental stages (19). To gain deeper insight into the developmental pattern of innervation associated with centrifugal renal arterial maturation and renin cell differentiation, we examined kidneys from E17.5, P0, P5, P10, and 1-month-old *Ren1^c-tdTomato/+^* mice (Figure 4A). We verified that the tdTomato signal is consistent with renin protein expression during development (Supplemental Figure 3, A and B). At E17.5 and P0, juxtamedullary AAs extending toward the medulla were clearly visible, and TUBB3^+^ nerve fibers had already reached and surrounded tdTomato^+^ renin-expressing cells along these arterioles. Synaptophysin^+^ pericellular nerve signals suggest the emergence of putative synaptic contacts (Supplemental Figure 3C). In agreement with the well-known centrifugal development — juxtamedullary nephrons develop earlier than cortical nephrons — of the kidney (19), tdTomato signals of renin-expressing cells in cortical vessels were initially (E 17.5 and P0) less prominent than in juxtamedullary AAs (Figure 4, A and B). Nevertheless, nerve fibers extended peripherally along those developing vessels (Figure 4, A, C, and E, and Supplemental Video 5). By P5, bright tdTomato^+^ clusters appeared at AA tips that were connected to developing glomeruli and displayed characteristic striped tdTomato^+^ patterns along the αSMA^+^ walls. In peripheral arteries nerves continued to precede tdTomato signal (Figure 4A and Supplemental Figure 3B). At P10, tdTomato^+^ clusters of renin cells were present and innervated at terminal AA segments, namely the JGA, across the arterial tree with weaker stripes remaining along the arterioles (Figure 4, A, D, and E). By 1 month, tdTomato^+^ renin cell clusters mainly located at JGA (Figure 4A). In summary, our 3D imaging analysis revealed distinct developmental dynamics between the inner and outer cortex, in which nerve fibers track along developing vascular walls and in contrast to the pattern observed in the inner cortex, are observed prior to robust renin expression in vascular wall cells.

### Stage-specific expression of axon guidance and neurotrophic factors by Forkhead box protein D1–lineage (FoxD1-lineage) cells in kidney development.

**Figure 5:**
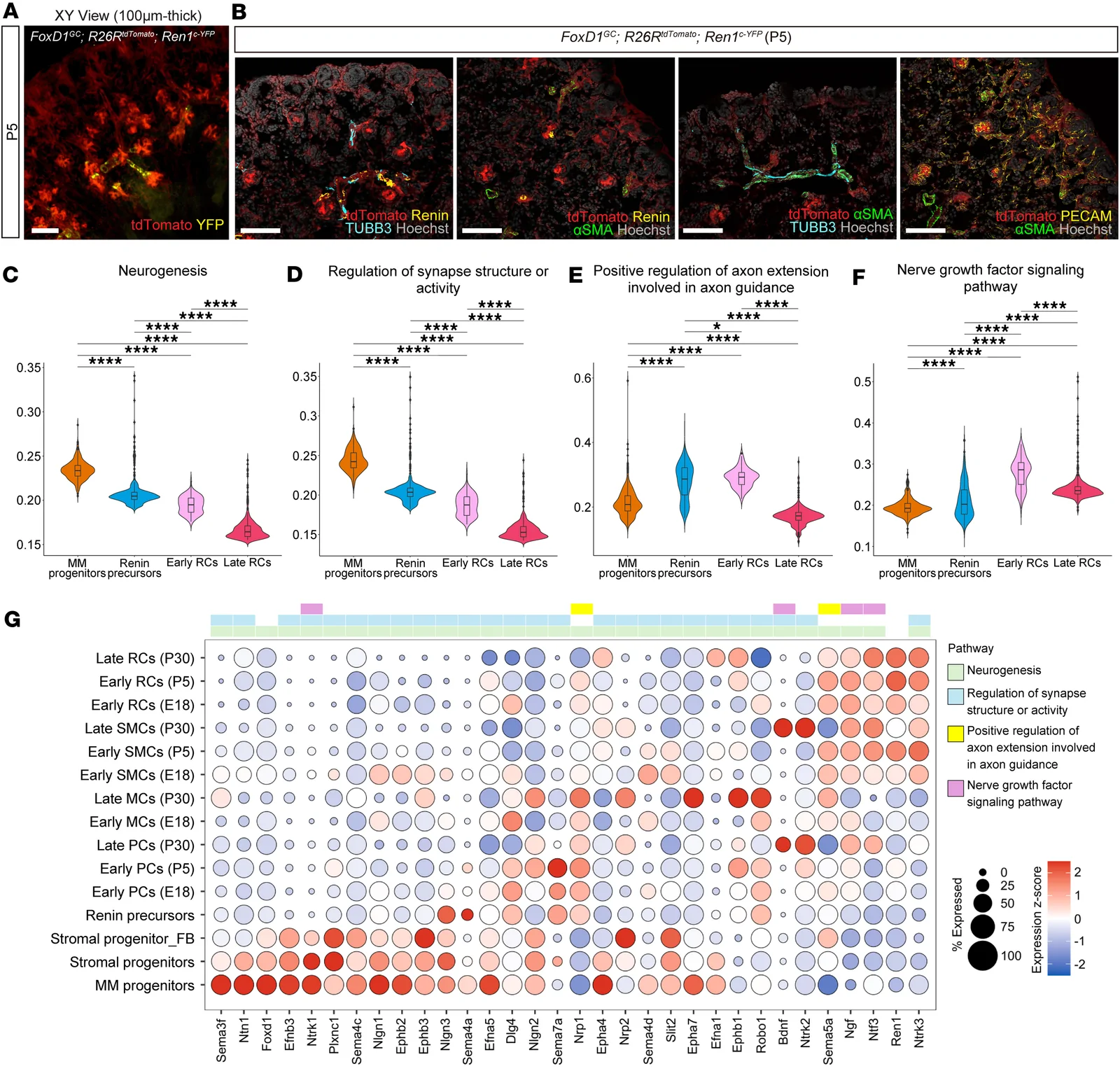
Stage-specific expression of axon guidance and neurotrophic factors by FoxD1-lineage cells during kidney development. (**A**) Representative XY-plane (100 μm thickness) 3D imaging of renal cortex from P5 *FoxD1^GC^; R26R^tdTomato^; Ren1^c-YFP^* mice, showing spatial distribution and relationship of renin-expressing cells (YFP) with FoxD1-lineage cells (tdTomato). Scale bar: 100 μm. (**B**) Immunofluorescence staining of P5 kidneys from *FoxD1^GC^; R26R^tdTomato^; Ren1^c-YFP^* mice illustrating the spatial relationships among renin cells, nerves, SMCs, and endothelial cells. Scale bars: 100 μm. (**C**–**F**) Violin plots showing cell-specific single pathway analysis scores for neurogenesis (**C**), regulation of synapse structure or activity (**D**), positive regulation of axon extension involved in axon guidance (**E**), and nerve growth factor signaling pathway (**F**) across MM progenitors, renin precursors, early renin cells (RCs), and late RCs. Statistical comparisons were performed using pairwise *t* tests with Benjamini-Hochberg multiple comparisons corrections; *P_adj_ < 0.05, ****P_adj_ < 0.0001. (**G**) Dot plot showing stage-specific expression patterns of selected nerve-related genes and cell markers (*Foxd1* and *Ren1*) across different populations of FoxD1-lineage renal cells at multiple developmental stages (E12, E18, P5, P30). Genes are color-coded by their associated GO pathways. Dot size: percentage of cells expressing the gene; color: expression levels (*z* score). MC, mesangial cell; PC, pericyte; FB, fibroblast. See also Supplemental Figure 4 and Supplemental Video 6.

Next, we investigated how immature vascular wall cells are distributed in relation to renin-expressing cells and nerves. During the development of the metanephric kidney, renin precursor cells originate from FoxD1-expressing stromal cells (2). These renin precursor cells subsequently differentiate into renin-producing JG cells, SMCs, mesangial cells, fibroblasts, and interstitial pericytes (18, 20, 21) (Supplemental Figure 4A). In *FoxD1^GC^; R26R^tdTomato^; Ren1^c-YFP^* mice, FoxD1^+^ cells are tdTomato^+^ and renin-expressing cells are YFP^+^. Kidneys from P5 mice clearly show tdTomato^+^ vessels (FoxD1 lineage) extending toward the developing glomeruli, and YFP^+^ cells (renin-expressing cells) were confined to AAs connected to mature glomeruli (Figure 5A and Supplemental Video 6). Immunofluorescence staining further confirmed immature FoxD1-lineage arterial walls formed along endothelial cells and that TUBB3^+^ nerve fibers advanced peripherally along FoxD1-lineage arterial walls and were closely associated with αSMA^+^ regions during outer cortical development (Figure 5B).

Then, to elucidate whether FoxD1-lineage cells participate in nerve-related signaling throughout kidney development, we utilized single-cell RNA-seq (GSE218570), derived from kidneys of *FoxD1^GC^; R26R^mTmG^* mice at E12, E18, P5, and P30 (Supplemental Figure 4, B and C) (22, 23). We applied Single Pathway analysis in Single Cells (SiPSiC) (24) and visualized pathway-activation scores across distinct cell populations, including metanephric mesenchyme (MM) progenitors, renin precursors, early renin cells, and late renin cells (Figure 5, C–F). Gene ontology (GO) terms indicated that FoxD1-lineage cells contribute to the stepwise establishment of renal vascular innervation across developmental stages: programs for neurogenesis and synapse structure/activity were enriched in early populations, axon-extension/axon-guidance modules peaked at intermediate stages (renin precursors and early renin cells), and NGF signaling rose with differentiation, emerging in early renin cells and remaining elevated in late renin cells. A dot plot of nerve-related genes further supported the stage-dependent pathway programs identified by GO analysis, revealing a developmental shift from axon-guidance cues toward neurotrophin-related expression during FoxD1-lineage differentiation (Figure 5G and Supplemental Figure 4D). Undifferentiated FoxD1-derived progenitor cells strongly expressed axon guidance cues, including *Sema3f*, *Ntn1*, *Efnb3*, *Plxnc1*, *Sema4c*, *Nlgn1*, *Ephb2*, *Ephb3*, *Nlgn3*, *Sema4a*, and *Efna5*, suggesting their role in guiding nerve fibers to precise locations within the developing renal tissue. Renin precursor cells were characterized by high expression of *Sema4a*, known as a neuroimmune semaphoring (25). Moreover, differentiated renin cells and SMCs showed elevated expression of neurotrophins, such as *Ngf*, *Ntf3*, and *Bdnf,* suggesting their role in stabilizing and maintaining innervation. Intriguingly, *Ngf* displayed an inverse expression pattern compared with its receptor *Ntrk1*, implying that NGF acts primarily as a paracrine signal toward adjacent nerves. Indeed, *Ntrk1(TrkA)*-KO mice have been reported to exhibit reduced renal innervation (26). As renin cells serve as major sympathetic targets in the kidney, our results also imply that renin-lineage cells contribute to sympathetic axon elongation, survival, and maintenance via the NGF/Ntrk1 axis. Conversely, coordinated expression of Bdnf/Ntrk2 and Ntf3/Ntrk3 receptor-ligand pairs supports autocrine signaling or reciprocal interactions within vascular mural cells, potentially contributing to vascular remodeling and neural plasticity. Notably, *Bdnf* and *Ntrk2* expression was highly enriched in mature SMCs and pericytes but was nearly absent in mature renin cells, suggesting a specific role for the Bdnf/Ntrk2 pathway in establishing and maintaining vascular mural cell identity. In summary, FoxD1-lineage cells sequentially contribute to kidney neural network formation, transitioning from roles in early neurogenesis and axon guidance during initial differentiation, to stabilization and maintenance of neural networks during later maturation stages.

### Renin cell–specific Ngf KO diminishes renin-lineage cells and their innervation.

**Figure 6:**
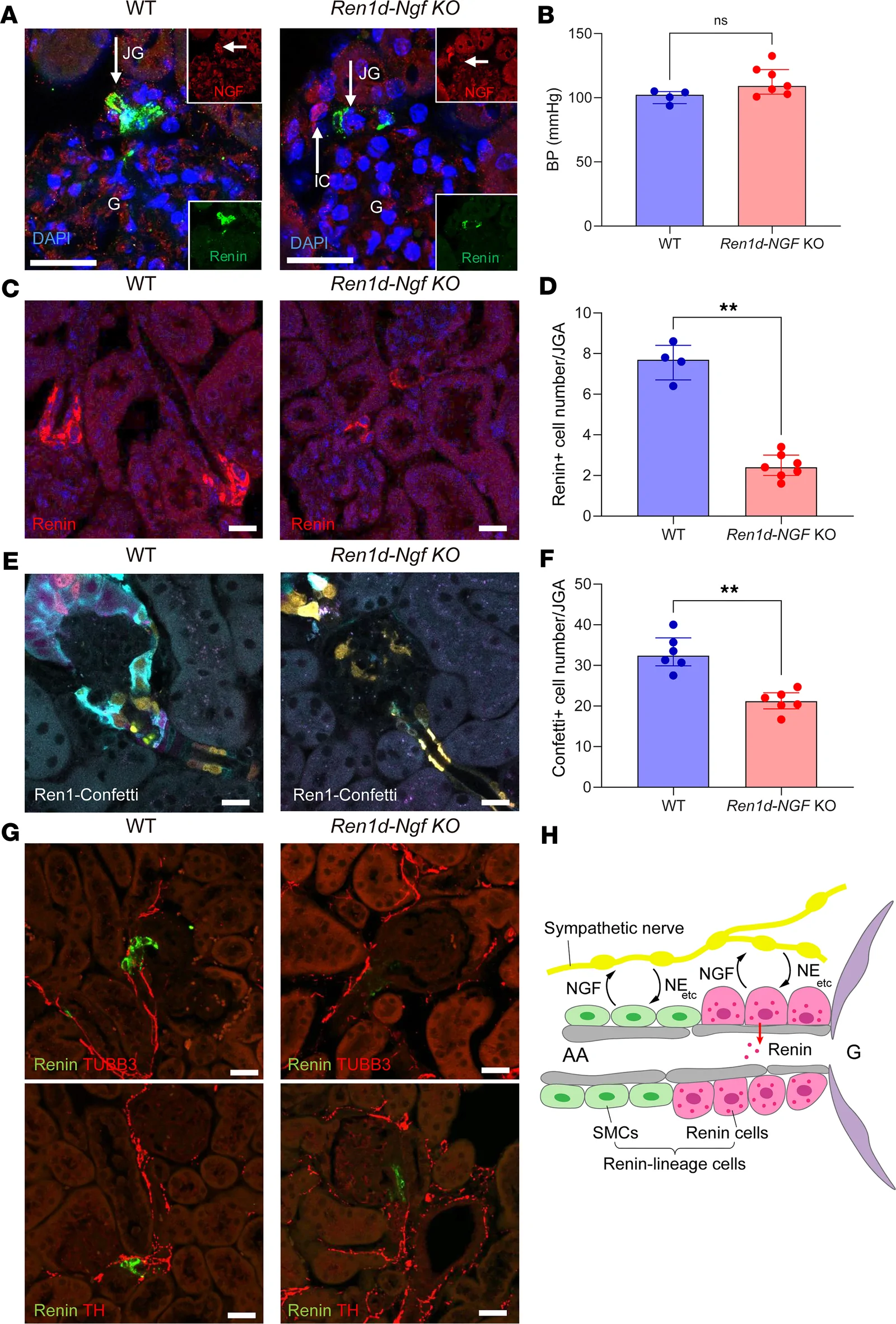
Renin cell–specific *Ngf* KO diminishes renin-lineage cells and their innervation. (**A**) Double immunofluorescence for renin and NGF in WT and *Ren1d-Ngf*–KO kidneys. Colocalization of renin and NGF is absent in *Ren1d-Ngf* KO. Scale bar: 25 μm. (**B**) Blood pressure in WT (*n* = 4) versus *Ren1d-Ngf*–KO (*n* = 7) male mice (Mann-Whitney *U* test). (**C**) Representative confocal images of renin immunofluorescence in WT and *Ren1d-Ngf* KO. Scale bar: 25 μm. (**D**) Quantification of renin^+^ cells per JGA in WT and *Ren1d-Ngf*–KO male mice (Mann-Whitney *U* test). (**E**) Representative confocal images showing Confetti-labeled renin-lineage cells in WT and *Ren1d-Ngf* KO. Scale bar: 50 μm. (**F**) Quantification of Confetti^+^ cells per JGA in WT and *Ren1d-Ngf*–KO male mice (Mann-Whitney *U* test). (**G**) Representative confocal images of TUBB3 (upper) and TH (lower) immunolabeling in WT and *Ren1d-Ngf* KO. Scale bar: 50 μm. (**H**) Concept of a coinductive feed-forward interaction between renin-lineage cells and sympathetic nerves. Renin-lineage cells secrete NGF to maintain sympathetic innervation, whereas sympathetic nerves release norepinephrine (NE) to stimulate renin synthesis and secretion by renin cells. Beyond NE, sympathetic nerves may also influence the renin-lineage cell phenotype through additional cotransmitters and trophic signals. **P < 0.01.

Next, to assess the functional significance of renin cell–derived NGF for renin cells and for sustaining cellular innervation, we generated a renin cell–specific *Ngf* KO by crossing *Ren1*^d-Cre/+^, *Ngf^fl/fl^*, and the multicolor reporter Confetti (*Ren1^d-Cre/+^; Ngf^fl/fl^; Confetti*; hereafter *Ren1d-Ngf* KO). Double immunofluorescence for NGF and renin confirmed selective loss of NGF in renin-lineage cells of *Ren1d-NGF*–KO kidneys, while NGF expression in collecting-duct intercalated cells (IC) was preserved as an internal positive control (Figure 6A). Blood pressure did not differ significantly between WT and *Ren1d-Ngf*–KO mice (Figure 6B). In the kidney, however, IHC showed fewer renin^+^ cells per JGA in *Ren1d-Ngf*–KO mice, corroborated by a reduced number of Confetti-labeled renin-lineage cells per JGA (Figure 6, C–F). The size of confetti-labeled arteriolar vascular SMCs cells and the intravascular diameter were reduced, indicating that specific *Ngf* KO affects the size and number of renin-lineage cells themselves. Finally, pan-neuronal (TUBB3^+^) and sympathetic (TH^+^) fibers avoided the renin cell region in *Ren1d-Ngf*–KO mice, in contrast to dense renin cell innervation in WT (Figure 6G). In summary, NGF from renin-lineage cells regulates the development and stabilization of normal sympathetic innervation and the number and size of renin-lineage cells. These findings support a feed-forward mechanism in which renin cells and axons coinduce one another to drive neurovascular development and maintain neuroendocrine function (Figure 6H).

### Renin enzymatic insufficiency leads to overstimulation of renin cells, and aberrant vascular hyperinnervation.

**Figure 7:**
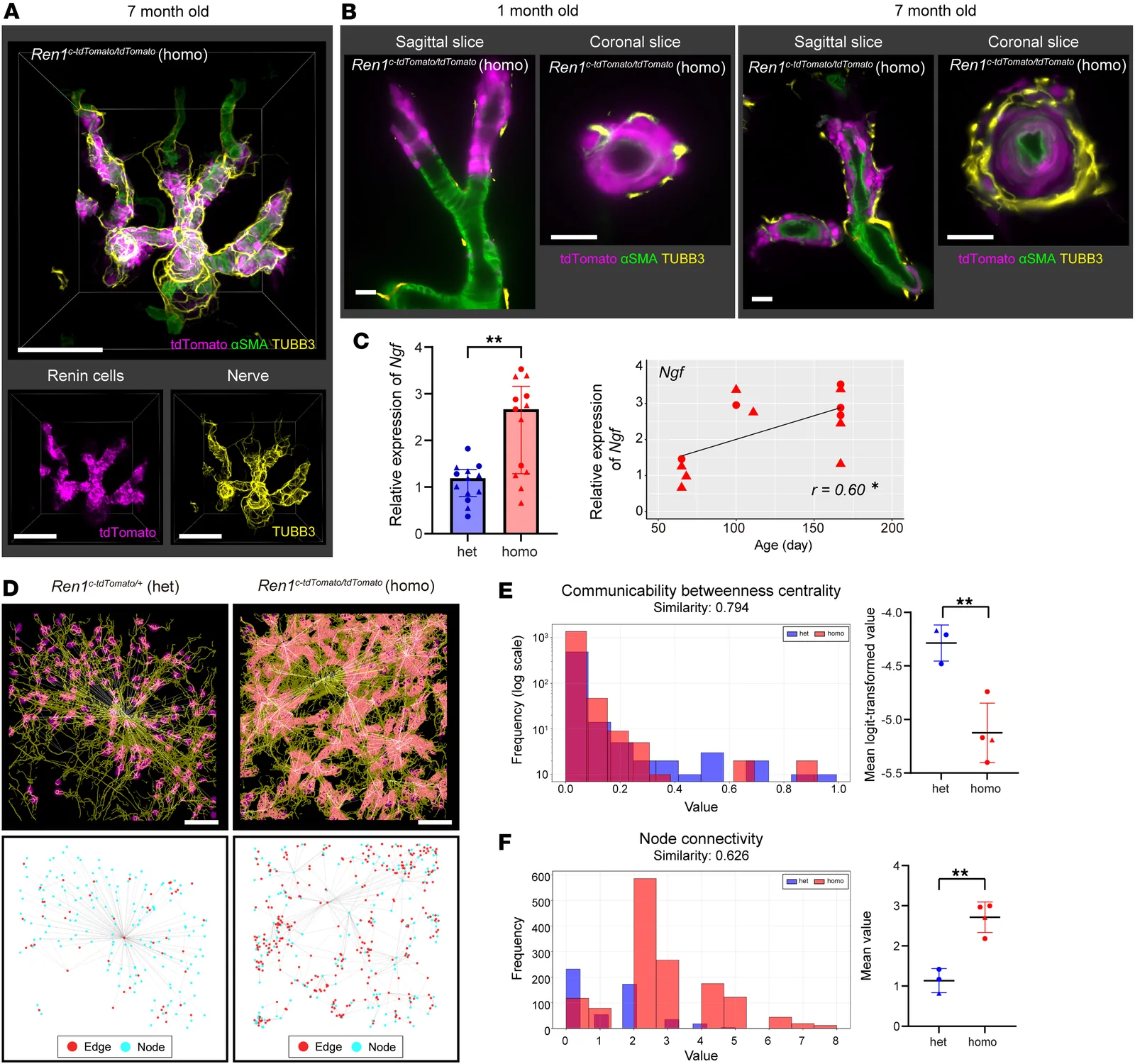
Renin enzymatic insufficiency leads to overstimulation of renin cells, NGF synthesis, and aberrant vascular hyperinnervation. (**A**) Representative 3D reconstructions of renin cells (tdTomato), vascular mural cells, and nerve fibers in kidneys of *Ren1^c-tdTomato/tdTomato^* (homo) mice at 7 months. Renin cells exhibit concentric expansion and progressive hyperinnervation. Lower panels separately show renin cells and nerve fibers. Scale bars: 100 μm. (**B**) Sagittal and coronal sections from 3D imaging illustrate progressive concentric thickening of AAs and increased perivascular innervation in homo kidneys at 1 and 7 months. Coronal views highlight lumen narrowing and the increasing complexity of nerve fiber organization. Scale bars: 20 μm. (**C**) qPCR for *Ngf* in kidneys from *Ren1^c-tdTomato/+^* (het) and homo mice at 2–5 months (Mann-Whitney *U* test, het *n* = 13, homo *n* = 13) (left). Pearson’s correlation between age and *Ngf* expression in homo mice (*n* = 13) (right). (**D**) Representative 3D reconstructions of renin-plexus networks generated with NetTracer3D from 7-month-old het and homo kidneys. Nodes represent renin cell clusters and nerve/plexus structures; edges indicate connections defined by an unbroken segmented nerve path between nodes. Scale bars: 200 μm. (**E** and **F**) Communicability betweenness centrality (**E**) and node connectivity (**F**). Overlaid histograms (left) show node-level distributions for representative networks. Group comparisons (right) show per-animal means analyzed by a linear mixed-effects model (het *n* = 3, homo *n* = 4); data are shown as mean ± SD; circles denote females and triangles denote males. Each histogram also reports a distribution-shape similarity score (0–1; higher indicates more similar), derived from the Jensen-Shannon distance. Triangles, males; circles, females, *P < 0.05, **P < 0.01. See also Supplemental Figures 5 and 6, and Supplemental Videos 7 and 8.

We next asked how this developmental neurovascular program behaves under chronic perturbation of renin signaling. Genomic or pharmacological ablation of the RAAS leads to a severe form of renal arterial disease characterized by the progressive thickening of the renal arterial tree (7–10). In this study, we employed the *Ren1^c-tdTomato^* mouse, whose renin cells respond properly to stimuli that elicit renin secretion and retain the capacity to synthesize renin and td-Tomato simultaneously, although the renin produced is enzymatically inefficient. Because heterozygous (*Ren1^c-tdTomato/+^*) animals have a normal renin allele, they have a normal phenotype, but homozygous (*Ren1^c-tdTomato/tdTomato^*) mice exhibit a distinctive defect: impaired renin enzymatic activity and reduced angiotensin generation (15). Renin cells attempt to compensate and are chronically stimulated to produce large amounts of an enzymatically inefficient renin enzyme. As a result, the kidneys develop progressive arteriolar hypertrophy and secrete large amounts of renin that is insufficient to maintain normal blood pressure (15). By 6 months of age, homozygous mice develop hypotension, reduced total glomerular filtration rate, and elevated BUN (15). Based on the hypothesis that chronic stimulation of renin cells in renal arterioles leads to hyperinnervation (10), we adapted the *Ren1^c-tdTomato^* mouse model for 3D image analysis. In 7-month-old *Ren1^c-tdTomato/tdTomato^* mice, we found concentric accumulation of renin cells along the renal arterial tree and hyperinnervation (Figure 7A and Supplemental Video 7). tdTomato^+^ renin cells adopted a bobbin-like, concentric layering around SMCs. Nerve fibers expanded along thick arterial walls and sent numerous branches juxtaposed to renin cells and the tortuous AAs. The nerve fibers ultimately enveloped the glomeruli. This hyperinnervation was predominantly TH^+^ sympathetic and synaptophysin^+^, consistent with en passant development of varicosities and synaptic connections (Supplemental Figure 5, A–C). The vascular disease progressed with age, showing a concentric buildup of renin cells, an inner αSMA^+^ SMC layer at the luminal border that further narrowed the vessels, and nerve fibers interwove among the renin-cell layers (Figure 7B and Supplemental Video 8). The relative mRNA abundance of *Ngf* was significantly higher in the kidneys of 2- to 5-month-old homozygous (Homo; *Ren1^c-tdTomato/tdTomato^*) mice compared with age-matched heterozygous (Het; *Ren1^c-tdTomato/+^*) control mice and exhibited a significant positive correlation with age and arteriolar disease (Figure 7C).

To further characterize the architecture of renin cell hyperinnervation, we performed 3D analyses of kidneys from 6- to 7-month-old Het and Homo mice using NetTracer3D, an algorithm optimized for renal neurovascular network reconstruction (27). We modeled a combined renin–plexus graph in which renin clusters and nerve/plexus structures were treated as nodes, connected by edges wherever an unbroken nerve path linked them (Figure 7D and Supplemental Figure 6, A and B). This makes visible the plexus-mediated wiring that links renin clusters through the surrounding neural architecture. In addition to trunk-aligned connectivity along the vascular tree, we also observed nerve trajectories linking renin clusters across different vascular territories in both Het and Homo kidneys (Supplemental Figure 6C).

Across the combined renin-plexus graphs, Het kidney networks showed higher communicability betweenness, consistent with a more efficiently organized communication structure (Figure 7E). In contrast, Homo kidney networks showed higher node connectivity, indicating increased local redundancy, which may reflect a more locally complex/entangled architecture rather than globally optimized integration (Figure 7F). Eigenvector centrality further supported this interpretation, with Het showing a clearer hub-like pattern and Homo displaying a more dispersed distribution of high-centrality nodes (Supplemental Figure 6D). Radial distribution, reflecting the physical distances spanned by network edges, was also consistent with a more locally dominated organization in Homo mice (Supplemental Figure 6E). Together, these results indicate that Het networks are more efficiently organized around defined hub-mediated communication pathways, whereas Homo networks show locally redundant wiring with more short-range connectivity and a more dispersed distribution of influential nodes — features that may alter signal propagation and coordination within the renal cortex.

In summary, under conditions of renin enzymatic insufficiency, the concentric accumulation of renin cells is accompanied by elevated NGF levels in the cortex, reinforcing renin cell–nerve interactions and contributing to a remodeling response prominently characterized by aberrant axon sprouting and sympathetic hyperinnervation.

## Discussion

**Figure 8:**
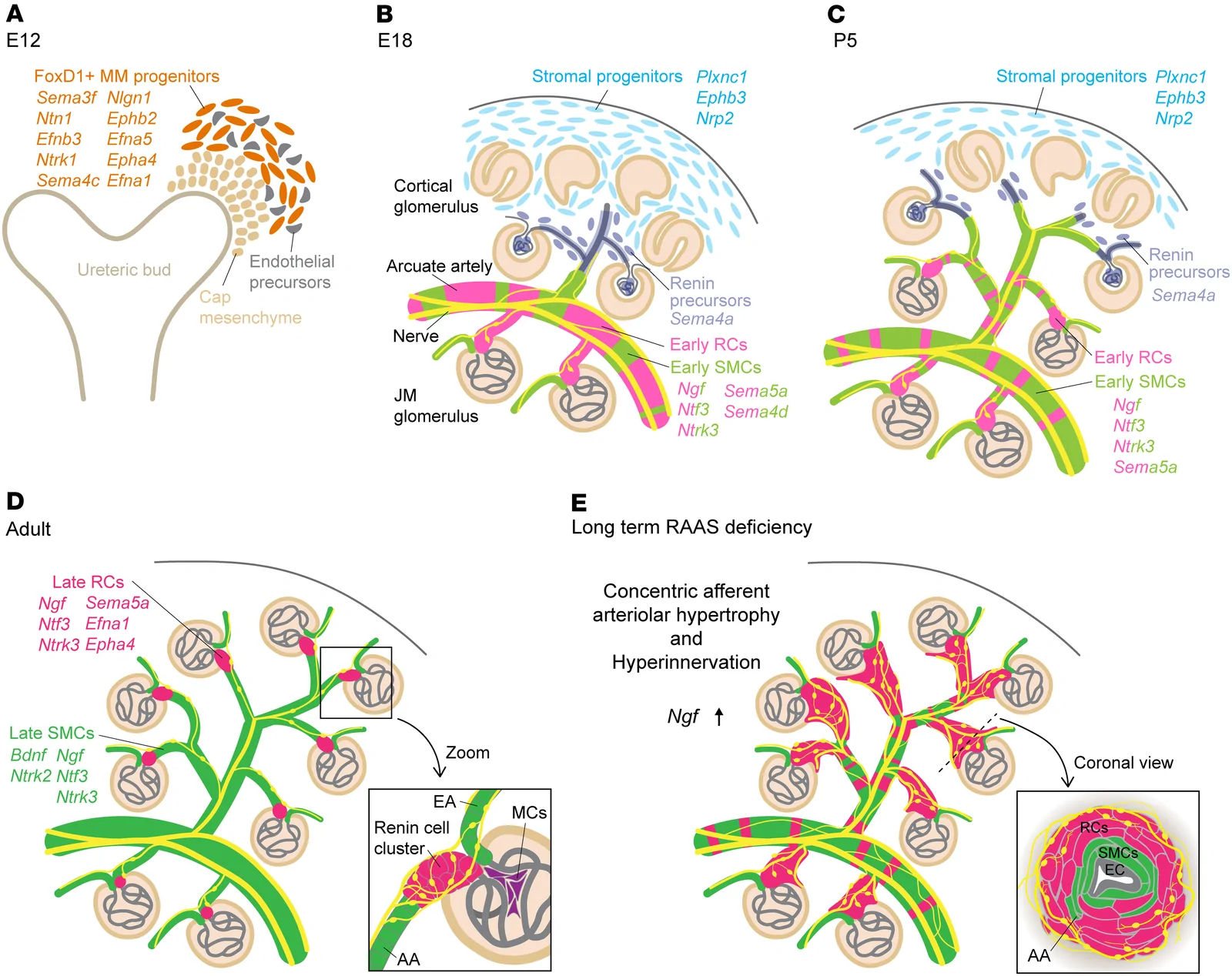
Schematic illustrating the spatiotemporal relationship between renin-cell distribution, arterial maturation, and axonal growth during normal development and renin enzymatic deficiency. (**A**) At E12, FoxD1^+^ MM progenitors express axon guidance molecules involved in early neurovascular patterning. (**B**) At E18, stromal progenitors, renin precursors, early renin cells (RCs), and early SMCs coordinate arterial formation and innervation. Early RCs/SMCs highly express neurotrophic factors such as *Ngf* and *Ntf3*, which stabilize and maintain tissue innervation. Arcuate arteries are extensively covered by RCs. Juxtamedullary afferent arterioles (JMAAs) and arcuate arteries are extensively covered by RCs, with nerves extending along them. In contrast, nerve fibers extend ahead of (and prior to) the vascular wall cells in cortical arteries. (**C**) At P5, early RCs diminish in arcuate arteries but remain prominent in JMAAs. In the cortex, early RC clusters emerge at cortical AA tips connected to developing glomeruli, forming striped patterns along arterial walls. In peripheral cortical regions, nerves extend before RC differentiation. (**D**) In adults, mature renin cells (late RCs) localize exclusively at juxtaglomerular regions, prominently in cortical glomeruli compared with JM glomeruli. Late RCs continuously express neurotrophic factors (*Ngf*, *Ntf3*, *Ntrk3*, *Sema5a*), and axon guidance molecules (*Efna1*, *Epha4*) reemerge at this stage. Late SMCs prominently express Bdnf and Ntrk2, alongside Ngf, Ntf3, and Ntrk3. Collectively, these expression profiles suggest cooperative roles of late RCs and SMCs in maintaining neurovascular structural integrity. Nerves branch into fine terminals forming effector junctions with RCs and extend toward efferent arterioles without innervating mesangial cells (MCs). (**E**) Long-term RAAS deficiency incudes concentric afferent arteriolar hypertrophy and hyperinnervation, associated with increased expression of *Ngf*, which likely contributes to structural remodeling of the renal vasculature and neural network. The coronal view illustrates concentric accumulation of SMCs, enlarged RCs encroaching upon the vascular wall, and proliferating nerve fibers, resulting in marked lumen narrowing. EC, endothelial cells.

In this study, we visualized with exquisite detail the rare renin cell in its “habitat” without disrupting the complex architecture of the kidney. Furthermore, we show the dynamic interactions of renin cells with other cells in the arterioles, glomeruli, and nerve fibers during healthy development, physiological challenges, and arterial disease in a model of renin enzymatic insufficiency (Figure 8). Here, we propose that renin cells are not merely responders to sympathetic input but also active organizers of their neuroregulatory microenvironment.

Due to their scarcity (0.01% of the total kidney cell mass), dynamic distribution, and complex spatial interactions with glomeruli and macula densa, renin cells present unique challenges for conventional 2D histological imaging. To overcome those limitations, we utilized the *Ren1^c-tdTomato^* mouse model, which express an ultrabright fluorescent reporter, tdTomato, under the endogenous renin gene locus (15). Combining this model with tissue clearing allowed unprecedented, in-depth and fine details of the renin cell morphology, localization, and cell-cell interactions throughout development and in response to physiological challenges and pathological stimuli. The study substantially deepens our knowledge largely obtained through 2D histological assessments (9, 28) and microdissections of the renal arterial tree (17). For instance, in the latter, we classified the distribution of renin cells within AAs into 7 distinct types that were corroborated by the present study, but the proportion of AAs totally lacking renin cells (Type 5) was over 30% in the previous study (17). Applying such classification to our 3D data, we found that only 2.5% of AAs in adult mice lacked renin cells under normal conditions. These findings illustrate the dramatically improved detection efficiency achieved with the newer methodology (2, 29). Furthermore, when an adult animal is exposed to a homeostatic threat, SMCs, MCs, and interstitial pericytes reenact the memory of the renin phenotype and are transformed into renin-producing cells. Here, we provide high-resolution 3D evidence of this phenomenon. During a short-term physiological challenge such as sodium depletion and captopril treatment, the recruitment of renin cells is characterized by an increased in volume (and number) of individual renin cells leading to hypertrophy of each JG cell cluster. This is also accompanied by the transformation of SMCs along the arterioles into renin-producing cells. The underlying mechanism involves chromatin remodeling and rapid epigenetic changes (1, 2, 30), without cell proliferation or migration (31). Interestingly, each renin cell increases its volume, likely reflecting enhanced synthetic activity and expanded organelle compartments, including renin granules, endoplasmic reticulum, Golgi, and mitochondria — typical markers of increased renin synthesis and release (1, 2, 28). Changes in ionic and water flow may also contribute but remain unexplored.

Furthermore, 3D imaging allowed us to examine the global — and detailed — spatial relationships between renin cells, and their surrounding nephrovascular and glomerular structures. The ability to see the whole while preserving the topology and connectivity between structures permitted us to determine that the arteriolar hypertrophy described above — and also discussed below — it is global in nature and, therefore, potentially accessible to a renal biopsy.

Accurate anatomical and functional maturation of neurovascular networks requires coordinated vessel-nerve interactions mediated by axon-guidance molecules and neurotrophins (32, 33). FoxD1-derived progenitors initially express axon-guidance cues that support early neural guidance and later shift to neurotrophins expression to stabilize mature neural connections (Figure 8). Importantly, genetic modification of renin lineage cell–specific *Ngf* KO confirmed that NGF produced by renin cells sustains cellular sympathetic innervation and is required to maintain renin cell development, abundance, and size. Together, these findings support a positive-feedback loop in which renin cells both receive and preserve the neural control that governs renin production.

Recently, 3D analysis of human renal neural networks revealed a network of adjacent glomerular clusters connected via “mother glomeruli” that serve as communicating hubs interconnecting glomerular communities throughout the cortex (27). This suggests that signals are sensed or relayed across nephrons and synchronized semiautonomously across the renal cortex. This neural network traverses the JGA. In this study, a similarly interconnected network of adjacent renin cell clusters was extracted. This implies that the regulation of renin synthesis and secretion is also integrated into this synchrony, suggesting that systemic blood pressure and homeostasis are controlled through communication among JG clusters serving interconnected nephrons. However, the organizer function of renin cells may, under chronic renin cell stimulation, markedly alter the local neurovascular environment in parallel with arteriolar hypertrophy. Sympathetic innervation density is typically correlated with target-derived NGF levels (34, 35). Under renin enzymatic insufficiency, neural network organization became more locally dominated and redundant around renin clusters, together with elevated *Ngf* expression levels in renal cortex (Figure 6). These changes in neural network architecture strongly suggest that excessive innervation preferentially targets and stimulates hyper-stimulated renin cells. This, in turn, may disrupt the synchrony maintained by the well-organized, mother-glomerulus–mediated internephron network and lead to network dysfunction. This concept is consistent with the notion that, under prolonged RAAS inhibition, the kidney eventually transforms into an neuroendocrine organ dedicated to the secretion of renin (10) at the expense of kidney function. The NGF-mediated positive-feedback loop between renin cells and sympathetic nerves that sustains renin cell function is further accelerated under RAAS inhibition, thereby synergistically promoting vascular remodeling and the overproduction of renin, likely representing a key driver of the pathological process. Importantly, as this remodeling progresses, it leads to decreased glomerular perfusion, impaired blood flow autoregulation, and an eventual decline in glomerular filtration rate (36). Unfortunately, the arterial disease remains undetected in clinical indices such as glomerular filtration rate until the pathology has become severe (36). Renal denervation may be partially effective in drug-resistant hypertension by resetting maladaptive renal neural networks that have undergone subclinical remodeling. On the other hand, if the stimulus to the renin cells persists, they will continue to act as active organizers, modifying the peripheral environment and driving vascular remodeling. The long-term safety of prolonged use or excessive blood pressure reduction through early intervention with RAAS inhibitors in blood pressure management should be evaluated considering histological changes due to renal neurovascular remodeling.

In conclusion, our study provides a high-resolution map of the dynamic spatial relationships between renin cells, renal vasculature, and sympathetic innervation across developmental and disease states. Our findings position renin cells as targets and drivers of the arterial sympathetic innervation that enables neural control of renin production and vascular development in health and disease. These findings advance our understanding of renovascular biology and the close coinductive interactions between the endocrine and nervous systems, and they offer potential avenues for the management of patients with cardiovascular, kidney and endocrine disorders.

## Methods

Supplemental Methods are available online with this article.

### Sex as a biological variable

Our study examined male and female animals, and similar findings are reported for both sexes.

### Animals

To label renin-expressing cells with fluorescent tdTomato under the control of the endogenous renin gene environment, we used *Ren1^c-T2A-tdTomato^* (*Ren1^c-tdTomato^*) mice generated in our laboratory (15). Heterozygous mice are indicated as *Ren1^c-tdTomato/+^*, and homozygous mice are indicated as *Ren1^c-tdTomato/tdTomato^*. To track the fate of renin cells and their descendants, we used *Ren1^c+/–^; Ren1^c-Cre^; R26R^mTmG^* mice generated in our laboratory as previously reported (8). In this line, renin lineage cells express GFP following Cre-mediated recombination, while nonrecombined cells retain RFP expression. To study the gene repertoire that characterize the earliest precursors of the kidney vasculature, including renin cells, we generated *FoxD1^GC^; R26R^tdTomato^; Ren1^c-YFP^* mice crossing *FoxD1^GC^* mice (Jackson Laboratory, 012463, RRID:IMSR_JAX:012463), *R26R^tdTomato^* mice (Jackson Laboratory, 007909, RRID:IMSR_JAX:007909), and *Ren1^c-YFP^* mice generated in our laboratory as previously described (37). To define the role of NGF in renin cell innervation and development, we generated renin cell–specific *Ngf* knockout mice (*Ren1d-Ngf*–KO) by crossing *Ren1*^d-Cre/+^ mice provided by our laboratory with *Confetti^fl/fl^* reporter mice (Jackson Laboratory, 013731, RRID: IMSR_JAX:013731) (38) and *Ngf^fl/fl^* mice (Liliana Minichiello, University of Oxford, Oxford, UK) (39). All animals used in this study were housed in a temperature- and humidity-controlled room under a 12-hour light/12-hour dark cycle. All animals used in this study were maintained in the C57BL/6 background. In WT and *Ren1d-Ngf*–KO mice, systolic blood pressure was measured using tail-cuff plethysmography as described earlier (40).

### Short-term captopril treatment with low salt diet

We treated 3-month-old Ren1c-tdTomato/+ (Het) mice ad libitum with captopril added to the drinking water (0.5 g/L) and a low sodium (0.1% Na^+^) diet for 8 days.

### Processing of mouse kidneys

Mice were anesthetized with tribromoethanol (300 mg/kg). Kidneys were removed and fixed overnight in formalin solution at room temperature (RT) and embedded in paraffin. For frozen sections, kidney tissues from *Ren1^c-tdTomato^*, *FoxD1^GC^; R26R^tdTomato^; Ren1^c-YFP^* and *Ren1d-Confetti* mice were fixed in 4% paraformaldehyde (PFA) for 2 hours at 4°C. After washing, tissues were cryoprotected in 30% sucrose suspended in PBS overnight at 4°C, embedded in optimal cutting temperature compound (OCT; Thermo Fisher Scientific), and subsequently frozen at –80°C.

### Immunofluorescence staining

Immunofluorescence staining was performed on 8 μm cryofixed sections or formalin-fixed, paraffin-embedded (FFPE) sections. FFPE sections were deparaffinized and rehydrated. For antigen retrieval, heat-induced epitope retrieval with Tris-EDTA (pH 9.0) was applied. After blocking with 5% (v/v) normal donkey serum (NDS) and 3% (w/v) BSA in PBS, sections were incubated overnight at 4°C with primary antibodies. Following washing and reblocking with 5% (v/v) NDS and 3% (w/v) BSA in PBS, the sections were incubated for 2 hours at RT with secondary antibodies. After further washes, sections were treated with an autofluorescence quenching kit (Vector Laboratories), counterstained with Hoechst 33342 (H3570, Thermo Fisher Scientific, RRID: AB_10626776) for nuclear visualization, and mounted in mounting medium (Vector Laboratories). The primary antibodies used were as follows: rabbit monoclonal anti-Renin antibody (1:5,000 dilution; ab212197, clone EPR20693, Abcam, RRID: AB_2909418), rat polyclonal anti-Renin antibody originally generated by Tadashi Inagami and colleagues at the Department of Biochemistry, Vanderbilt University School of Medicine (41) (1:5,000 dilution), goat polyclonal anti-Renin antibody (1:100, AF4277, R&D Systems, RRID: AB_2179475), mouse monoclonal anti-neuron–specific β-III tubulin (TUBB3) antibody (1:100, MAB1195, clone TuJ-1, R&D Systems, RRID:AB_357520), mouse monoclonal Alexa Fluor 647–conjugated anti-TUBB3 antibody (1:200 dilution; 657406, clone AA10, BioLegend, RRID: AB_2563610), rabbit polyclonal anti-TH antibody (1:200 dilution for cryo-fixed sections, 1:100 dilution for FFPE sections; AB152, Sigma-Aldrich, RRID: AB_390204), rabbit polyclonal anti-synaptophysin antibody (1:100 dilution; ab14692, Abcam, RRID: AB_301417), rabbit monoclonal Alexa Fluor 488–conjugated anti–CGRP-I+CGRP-II antibody (1:100 dilution; ab305115, clone EPR23804-95, Abcam), mouse monoclonal fluorescein isothiocyanate–conjugated (FITC-conjugated) anti-αSMA antibody (1:100 dilution; F3777, clone 1A4, Sigma-Aldrich, RRID: AB_476977), and goat polyclonal anti-platelet endothelial cell adhesion molecule 1 (PECAM-1) antibody (1:100 dilution; AF3628, Bio-Techne, RRID: AB_2161028), and rabbit polyclonal anti-NGF antibody (1:100, OSN00106W, Thermo Fisher Scientific). The secondary antibodies used were Alexa Fluor 488–conjugated donkey anti-rabbit antibody (1:400 dilution; A-21206, Thermo Fisher Scientific, RRID: AB_2535792), Alexa Fluor 488–conjugated donkey anti-goat antibody (1:400 dilution; A-11055, Thermo Fisher Scientific, RRID: AB_2534102), and Alexa Fluor 647–conjugated donkey anti-rabbit antibody (1:400 dilution; A-31573, Thermo Fisher Scientific, RRID: AB_2536183).

### Microscopy

Tissue sections from *Ren1^c-tdTomato^* and *FoxD1^GC^; R26R^tdTomato^; Ren1^c-YFP^* mice were imaged using a Zeiss Imager M2 microscope equipped with an Apotome.2 optical sectioning device fitted with AxioCam 305 color and AxioCam 506 mono cameras (Zeiss). Tissue sections from *Ren1d-Ngf*–KO mice were imaged using a Leica Stellaris 8 Falcon multimodal multiphoton microscope with Chameleon Discovery NX laser (Leica).

### Tissue clearing and whole-mount immunostaining of mouse kidneys

Mouse kidney tissue clearing and whole-mount immunostaining were conducted using an optimized version of the Clear, Unobstructed Brain/Body Imaging Cocktails and Computational Analysis (CUBIC) method (42). Procedures were based on previously described protocols (43, 44). Mice were anesthetized using tribromoethanol (300 mg/kg) and subsequently perfused through the left ventricle with 20 ml PBS, followed by 30 ml of 4% PFA. Kidneys were excised, bisected, and fixed overnight in 4% PFA. All subsequent procedures were conducted with gentle agitation. After fixation, samples were washed in PBS for 6 hours, followed by immersion in CUBIC-L solution (10 wt% N-butyldiethanolamine and 10 wt% Triton X-100 [MilliporeSigma]) at 37°C for 5 days. Samples underwent another 6-hour PBS wash and were blocked overnight at RT in blocking buffer (PBS containing 1.0% bovine serum albumin and 0.01% sodium azide). Next, samples were incubated in immunostaining buffer (PBS containing 0.5% Triton X-100, 0.25% bovine serum albumin, and 0.01% sodium azide) supplemented with fluorescently labeled antibodies: FITC conjugated anti-αSMA antibody (1:100 dilution, F3777, Sigma-Aldrich), Alexa Fluor 647–conjugated anti-TUBB3 antibody (1:200 dilution, 657406, BioLegend), Alexa Fluor 488–conjugated anti–CGRP-I+CGRP-II antibody (1:100 dilution, ab305115, Abcam), or TOPRO3 nuclear dye (1:1,000 dilution, T3605, Life Technologies), for 5–14 days at RT. Staining duration varied depending on sample size. After a subsequent 6-hour PBS wash, samples stained with anti-TUBB3 antibody or anti-CGRP antibody underwent postfixation in 1% PFA for 3 hours. All samples were then immersed overnight at RT in a 1:1 dilution of CUBIC-R^+^ solution (T3741 [containing 45 wt% 2,3-dimethyl-1-phenyl-5-pryrazolone, 30 wt% nicotinamide, and 5 wt% N-butyldiethanolamine], Tokyo Chemical Industry), followed by undiluted CUBIC-R^+^ solution immersion at RT for an additional 2 days.

### Light-sheet fluorescence microscopy

Cleared kidney samples were imaged using a Zeiss Lightsheet 7 fluorescence microscope (Zeiss). Specimens were mounted in a refractive index matching solution (RI = 1.520; M3294, Tokyo Chemical Industry) and visualized using Lightsheet 7 detection optics with either a 5×/0.16 foc or a Clr Plan-Neofluar 20×/1.0 Corr nd = 1.53 objective lens. Voxel resolution was as follows: for the 5× objective lens (zoom: 1.5), *x* = 0.632 μm, *y* = 0.632 μm, and *z* = 3.38–3.61 μm; for the 20× objective lens (zoom range: 0.36–2.0), *x* = 0.118–0.656 μm, *y* = 0.118–0.656 μm, and *z* = 0.549–1.00 μm. Excitation wavelengths were 488 nm for GFP, FITC, Alexa Fluor 488, or YFP; 561 nm for tdTomato; and 638 nm for Alexa Fluor 647 or TOPRO3 signals.

### Three-dimensional image

Three-dimensional reconstruction and analysis of captured images were performed using Imaris software (Version 10.0.0, Bitplane). Raw Zeiss microscopy data (.czi files) were converted to Imaris-compatible files (.ims format) using Imaris File Converter 10.0.0, with tile scan images stitched by Imaris Stitcher 10.0.0. Image processing in Imaris was performed according to established protocols (43, 44). Three-dimensional reconstructions were cropped into areas of interest using the crop function, and images or videos were generated using the snapshot and animation functionalities, respectively.

### Relative tdTomato intensity in juxtamedullary AAs (JMAA) and arterial branches from arcuate arteries to the outer cortex (AB-AAC) and Confetti image analysis

For the analysis in Figure 4D, tdTomato signal was quantified using the Surface module in Imaris. For each sample, several JMAA and AB-AAC were manually segmented as individual surface objects. For each surface, the maximum tdTomato intensity within the object was extracted, yielding one value per vessel. For normalization, the mean of the maximum tdTomato intensities of all JMAA vessels within each sample was calculated, and the maximum intensity of each vessel (JMAA or AB-AAC) was divided by this mouse-specific JMAA mean, such that the relative tdTomato intensity of JMAA was centered at 1.0 for each mouse. Fluorescence imaging of the multicolor Confetti reporter (CFP-GFP-YFP-RFP) (45) and the analysis of Confetti^+^ cell density and clonality (40) were performed as described recently.

### Distance profiling and Δd analysis

#### Background estimation and channel preprocessing.

For each sample, background intensity was estimated using the Surface module in Imaris (version 10.0.0). Using the manual circular selection tool, 5 small circular regions of interests were placed in tubular areas without specific signal, and 5 background surface objects were generated. For each of these surfaces, the maximum intensity of the tdTomato and TUBB3 channels was measured, and the mean of the 5 values was calculated to obtain the background intensity for each channel in that sample. A new set of surface objects was then generated for each channel using the sample-specific background intensity as the lower intensity threshold. Using the Mask function of the Surface module, we created background-subtracted tdTomato and TUBB3 channels for subsequent analysis.

#### Surface definition and distance profiling.

The kidney surface was segmented in Imaris using the Machine Learning Segmentation function of the Surface module. All channels were used as reference signals during training, with regions outside the tissue annotated as include and regions inside the tissue annotated as exclude, allowing the model to learn the boundary between the specimen and the surrounding background. The sample-facing side of the resulting outer-surface object was taken as the kidney surface. Using the Scatter Plot module, the mean intensity of the background-subtracted tdTomato and TUBB3 channels was extracted as a function of the distance from the surface in the outward direction, generating a distance–intensity profile for each sample.

#### Binning, onset detection, and Δd calculation.

Distance values were grouped into 5-μm bins, and the mean intensity of the background-subtracted tdTomato and TUBB3 channels within each bin was calculated to obtain distance-dependent intensity profiles. For each channel, the onset distance (d_onset) was defined as the first 5 μm bin in which the slope of the profile exceeded 0.01, where slope was calculated as (I_{i+1} − I_i)/5. The separation between channels was quantified as *Δd* = d_onset(tdTomato) − d_onset(TUBB3).

### Three-dimensional renin–plexus network analysis by NetTracer3D

To characterize the architecture of renin cell innervation, we used NetTracer3D (46), an open-source Python-based tool that converts volumetric imaging data into quantifiable network graphs (27). Image preprocessing of LightSheet 7 datasets was performed as previously described (27).

Segmentation of nerves and renin clusters was performed using labkit (47) and NetTracer3D. Renin cell clusters (tdTomato^+^) were treated as graph nodes. Euclidean distance transformation was utilized to search 12 μm from nodes, which partitioned the interacting nerves into segments “before” and “after” the search region. Edges were initially defined by continuity of the segmented nerve signal, such that 2 nodes were considered connected when an unbroken nerve path linked them. This resulted in an initial graph between renin clusters. To eliminate connectivity biases along nerve trunks, after initial renin network computation, the connecting nerve segments were converted to nodes as well. This merged both structures into a graph showing plexus-mediated wiring that links renin clusters through the surrounding neural architecture, rather than direct renin-to-renin contacts alone.

Graph-level and node-level metrics were computed in NetTracer3D using NetworkX (48), including communicability betweenness centrality, node connectivity, eigenvector centrality, and radial distribution (distribution of physical distances spanned by network edges). For visualization, networks were rendered both in 3D (preserving tissue coordinates) and as 2D spring-layout graphs; the spring layout places more strongly connected node groups closer together to facilitate visualization of network clustering and does not reflect physical spatial coordinates.

For group comparisons between *Ren1^c-tdTomato/+^* and *Ren1^c-tdTomato/tdTomato^* mice, per-animal mean metric values were analyzed using linear mixed-effects models. For node connectivity values bounded between 0 and 1, we applied a logit transformation prior to linear mixed-effects modeling. In histogram panels, a similarity score (0–1; higher indicates more similar distribution shape) was reported as Jensen-Shannon distance (49) subtracted from 1.

### Dot plot for single-cell RNA-seq analysis

Our previously published single-cell RNA-seq data from embryonic and adult *FoxD1^Cre^; R26R^mTmG^* mouse kidneys (GSE218570) (22, 23) were reprocessed to generate dot plots of neurogenesis-related genes in renin-lineage cells. The raw expression matrix was log-normalized, z-score transformed, and the percentage of cells expressing each gene was calculated across all cell populations in the gene expression matrix that composed the renin cell trajectory. The output from this function was then plotted using ggplot2 (50).

### Pathway enrichment analysis

To calculate single-cell enrichment of gene sets and pathways, we utilized annotated gene sets from the Molecular Signatures Database (51, 52) for mouse hallmark, regulatory target, curated, cell signature, biological process, cellular component, and molecular function gene sets. Gene sets were scored on individual cells using the SiPSiC (v.1.4.3) R package (24). Individual cell pathway scores for each cell population were plotted using ggplot2 (50). Significant differences were calculated by pairwise t tests with the Benjamini-Hochberg multiple testing procedure.

### Statistics

Statistical analyses were performed using GraphPad Prism version 10.4.1 (GraphPad Software). Unpaired comparisons were performed using 2-tailed Mann-Whitney *U* tests. Data are presented as median (interquartile range [IQR]). Linear mixed-effects models were fitted for 2 sets of comparisons: (a) vessel-type differences (JMAA versus AB-AAC), with vessel type as a fixed effect and mouse ID as a random intercept, and (b) genotype effects in the NetTracer3D analysis, with genotype as a fixed effect and mouse ID as a random intercept. For multiple comparisons, pairwise 2-tailed *t* tests with Benjamini-Hochberg correction was used. Pearson’s correlation coefficient was calculated for linear correlation analyses. *P* < 0.05 were considered statistically significant.

### Study approval

All procedures were performed in accordance with the *Guide for the Care and Use of Laboratory Animals* (National Academies Press, 2011) and were approved by the IACUC of the University of Virginia (protocol no. 2433) and the University of Southern California (protocol no. 10531).

### Data availability

The single-cell datasets analyzed in this study are publicly available through the NCBI Gene Expression Omnibus under accession no. GSE218570 (22). Scripts to reconstruct the annotated Seurat objects used as starting points for analysis are available at https://github.com/jpsmith5/renin_analysis (commit ID: dbc64226a23a90863671c6fde8d56830ade35cec). R scripts for SiPSiC pathway scoring (Figure 5, C–F) and neuronal gene expression visualization (Figure 5G) are available at https://doi.org/10.5281/zenodo.21038422 NetTracer3D is publicly available at https://pypi.org/project/nettracer3d/ (27, 46, 53). The Python script used for 3D morphological analysis is provided as Supplemental Data File 1. Values for all data points in graphs are reported in the Supporting Data Values file. Mice generated in our laboratory are available to the scientific community upon request.Materials availability.

## Author contributions

MLSSL and RAG designed the study and supervised the project. MY, GG, and HY performed experiments. MY, LM, HY, and KT developed methodology. SM developed mouse model. MY, GG, LM, HY, JPS, and SH analyzed the data. SMW performed EM. MY drafted the initial version of the manuscript. SJ, JPP, MLSSL, and RAG read, reviewed, redrafted, and edited the manuscript. GG, LM, HY, JPS, LFDA, DM, AGM, SMW, SH, KT, SM, SJ, JPP, MLSSL, and RAG reviewed and edited the manuscript. All authors approved its final version.

## Conflict of interest

KT is a coinventor on Japanese and international patents and patent applications covering CUBIC-related tissue-clearing technology. SJ and LM have an intellectual property invention disclosure on the 3D nerve network analysis in solid organs software tool NetTracer3D. The copyright of NetTracer3D is held by Washington University in St. Louis, and SJ and LM may receive royalties from commercial use. JPP and GG are cofounders of Macula Densa Cell LLC, a biotechnology company that develops therapeutics to target macula densa cells for a regenerative treatment for chronic kidney disease. Macula Densa Cell LLC has a patent entitled “Targeting macula densa cells as a new therapeutic approach for kidney disease” (US patent nos. 10,828,374 and 11,318,209).

## Funding support

This work is the result of NIH funding, in whole or in part, and is subject to the NIH Public Access Policy. Through acceptance of this federal funding, the NIH has been given a right to make the work publicly available in PubMed Central.

- NIH grants P50DK096373 and R01DK145876 to RAG and MLSSL.
- NIH grant R01DK116718 to RAG.
- NIH grant R01HL148044 to MLSSL.
- NIH grant P50DK133943 to SJ.
- NIH grant R01DK064324 and R01DK123564 to JPP.
- Japan Society for the Promotion of Science Overseas Research Fellowships (202470020) to MY.

## Supplementary Material

Supplemental data

Supplemental Data File 1

Supplemental video 1

Supplemental video 2

Supplemental video 3

Supplemental video 4

Supplemental video 5

Supplemental video 6

Supplemental video 7

Supplemental video 8

Supporting data values

## Version 1 07/21/2026

In-Press Preview

## Version 2 08/24/2026

Electronic publication
